# Proactive measures to eradicate Malaysia’s poverty in IR4.0 era: a shared prosperity vision

**DOI:** 10.12688/f1000research.73330.1

**Published:** 2021-10-28

**Authors:** Darshana Darmalinggam, Maniam Kaliannan, Magiswary Dorasamy

**Affiliations:** 1 Nottingham University Business School, University of Nottingham Malaysia, Semenyih, Selangor, 43500, Malaysia; 2 Faculty of Management, Multimedia University, Cyberjaya, Selangor, 63100, Malaysia

**Keywords:** Malaysia, poverty, equality, training needs analysis, multi-stakeholder, B40, youth, proactive

## Abstract

**Background:** In the country’s shared prosperity vision, Malaysia aspires to uplift the bottom 40% household income group (B40) by addressing wealth and income disparities. By 2030, the nation seeks to eradicate poverty through the provision of employment opportunities and career progression plans. A grey area between the nation’s aspirations and actions in practice can be observed because the goals have not been achieved despite numerous efforts aimed at the upliftment of the B40 group. The nation is still way behind its targeted outcomes despite various policies being implemented, which could be attributed to the mismatch between government policies and that of organisational practice. Thus, this study explores the rationale of strategic government intervention in managing B40 talent in the IR4.0 era.

**Methods**: A general qualitative inquiry method that used 11 semi-structured interviews was carried out with representatives of Malaysia’s policy makers’, training providers, and trainees. All Interview questions centred around measures, importance and outcomes of B40 youth training from a multi-stakeholder perspective. Data were thematically analysed in five stages using NVivo.

**Results:** Training, which includes IR4.0 era digital skills, is the key to uplifting the B40 youth to eradicate poverty. Proactive measures are imperative in the success of B40 youth training towards poverty eradication.

**Conclusions:** This study contributes to the existing literature and helps practitioners by addressing the current gap in Malaysia’s aspirations versus organisational practice. Stakeholders should formulate proactive strategies to ensure that the right trainees are matched with the right training providers and government policies. A linkage between government policies and industry requirements needs to be established as opposed to the present discontinuity. A structured training needs analysis should be applied through a collaboration between industries and governments. Then, B40 individuals commonly found in lower-level positions can be pooled into the career pathway towards a shift into M40.

## Introduction

In the country’s shared prosperity vision 2030, Malaysia aspires to uplift the bottom 40% household income group (B40). Accordingly, the shared prosperity vision aims to (1) develop all citizens in various levels through economic restructuring of full community participation towards a more progressive, knowledge-based and highly valued community, (2) address income and wealth disparities through eradicating inequalities and ensuring that no one is left behind and (3) attaining a united, prosperous and dignified nation through nation-building in becoming Asia’s economic centre. ^9^ By 2030, the nation seeks to eradicate poverty through the provision of employment opportunities and career progression plans. However, a grey area between the nation’s aspirations and actions in practice towards poverty eradication can be observed. ^1^ Poverty eradication in Malaysia refers to those in the B40 group. The goals have not been achieved despite numerous efforts aimed at the upliftment of the B40 group. The nation is still significantly behind its targeted outcomes despite various policies being formulated, which can be attributed to the mismatch between government policies and organisational practice. To re-match the two aspects, Malaysia could focus more attention on inclusive talent development practices targeted at the B40 youth. This inclusive youth training approach has been supported in past literature. ^10^ The B40 group represents the poverty that Malaysia is currently facing. Many of the B40 group households tend to have lower-level job positions or are unemployed because of mismatches or lack of the right knowledge, skills, abilities and attitude. The more relevant knowledge, skills and abilities in this industrial revolution (IR) 4.0 era include training programs that focus on data sciences, green accounting, and forensic economics. ^11^ Thus, to eradicate poverty in the long run, Malaysia should uplift the B40 youth group because they are the future generation that would determine the poverty level in Malaysia. Once they are uplifted into the middle 40% household income group (M40), poverty would be eradicated for good as they grow up to become adults or parents of the M40 group. Accordingly, this study aims to explore the rationale of government intervention in managing B40 youth talent via training in this IR4.0 era. The following research questions guide the study.

Research question 1: To what extent is training important in uplifting the B40 youth in line with poverty eradication?

Research question 2: What are the proactive measures and to what extent are they crucial to the success of B40 youth training towards poverty eradication?

## Methods

A general qualitative approach using interviews from an interpretative perspective was used in this study. ^2^ The researchers believe that the knowledge on proactive measures to uplift the B40 youth is interpreted from the results of the study. The entire study took place within 2 years (2019-2021). Details on the research design are provided in Table 1. The research idea was outlined by researcher 1, a master’s student, and researcher 2, a lecturer (associate professor), who has vast experience in teaching human resource management modules and has undertaken several other research projects concerning talent management. Researcher 3, also a lecturer (associate professor), is an NVivo software expert which was used for data analysis. However, the suitability of software usage was revisited post-data collection, using a methodological reflexivity approach. ^3^ Researchers 1 and 3 are females and researcher 2 is a male, and thus no gender bias is thought to be evoked throughout the study. Overall, the study was informed by the researchers’ understanding, knowledge and experience on the subject matter, reflecting ontological reflexivity to a certain degree.

**Table 1.** Qualitative research design used for the study.

Criteria	Description
Research paradigm	o Interpretative (Inquiry and investigation are prime method)
Method deployed	o General Qualitative Inquiry Method
Data collection techniques	o Interviews (multi-stakeholder groups)
Population	o Unit of analysis: Individuals o Respondents: Representatives from policy makers, trainers, and trainees o Sampling Method: Purposive Sampling Method
Data analysis	o Thematic Analysis – 5 Stages (become familiar with data, generate initial codes, search for themes, review themes, define themes) o Word Frequency Analysis, Word Cloud, Mapping of Themes to Con
Validity and Reliability	o Credibility – Research findings are plausible information drawn out from participants’ original data and is a correct interpretation of the participants’ original views. o Transferability – The researchers facilitates transferability judgement by a potential user through thick description. o Dependability – Participants’ evaluation of the findings, interpretation and recommendations of the study are all supported by the data received from participants. o Confirmability – Data and interpretations of the findings are not figments of the inquirer’s imagination, but clearly derived from the data. o Reflexivity – Researchers constantly self-reflected oneself as a researcher (own biases, preferences, preconceptions), and the research relationship (relationship to the respondents), and how the relationship affects participants’ answers to question

Purposive random sampling ^4^ was used to gain insights from three stakeholder groups namely policymakers, training providers and trainees to obtain a multi-stakeholder perspective on B40 youth upliftment via training. Training providers provide various trainings including entrepreneurial, electrical, computer, and wiring areas which mainly target Malaysian youth. Policymakers were approached via emails to the relevant ministries which then directed the researchers to representatives in charge. Only trainees who had surpassed six months post-training completion were approached through a training provider. Trainees approached underwent skill-based training programmes on various courses including computer, electrical and wiring areas. These B40 youth trainees were given monetary compensation for their time while representatives from the policymakers and training providers voluntarily participated in the study. All potential participants who were approached took part. A total of 11 participants were attained, surpassing the suggested sample size of 4 to 10 for case studies. ^5^ No prior relationship was established with participants. Approval for data collection was obtained from the University of Nottingham Malaysia ethics committee and Nottingham University Business School Research Ethics Committee (ref number: NUBS-REC-2021-11). All participants and respective higher authorities for each stakeholder group were orally asked for consent, their confidentiality guaranteed, and the interviews were only conducted upon their agreement. Oral consents as such are allowed under NUBS REC’s pre-approved protocol regulations.

One-on-one semi-structured online interviews were conducted between 15 April and 1 May 2021 by researchers 1 and 2. Semi-structured interviews were preferred because the study was exploratory. ^6^ Interview questions centred around measures, importance and outcomes of B40 youth training (refer to Table 2 for details). For example, details of training courses, benefits of training courses, challenges faced throughout training, and nation’s strategic plan on B40 youth training. Probe questions were catered towards specific group of stakeholders accordingly. The interview schedule can be found as *Extended data*. ^13^ Subsequently, data were transcribed and analysed using NVivo software (version released in March 2020) for a month. A pilot interview was conducted with the third researcher to ensure that the questions are clearly understood by the interviewees. No changes were made to the interview guide as deemed appropriate. Before the interviews, every interviewee was provided with a rough question guide on areas the researchers would cover in the interview. At the start of the interview session, participants were briefed on the researchers and the study area. Each interview session varied between 40 minutes to 1 hour and interviews were not repeated. All interviews were audio-recorded through Microsoft Teams or Google Meet platforms’ recording functions upon consent from interviewees. Occasionally, issues with the internet connection disrupted the flow of the interview sessions. Nevertheless, the researchers managed to cope with the situation and continue where they left off. The two applications were used to conduct the interviews based on interviewees’ preferences and comfort. Probing questions were used whenever the researchers needed to gain in-depth insights from the participants. Field notes were recorded during the online interview sessions as a backup and were cross-checked against transcriptions for accuracy. Details of the 11 interviewees can be found in the results section of the report.

**Table 2.** Interview questions asked for each research question and their respective target groups.

Research question	Interview questions	Target multi-stakeholder group
1) To what extent is training important in uplifting the B40 youth in line with poverty eradication?	o What is the success rate of the training like? o What are the benefits of training? o Do you agree that training has had a positive impact on your lives?	o Trainer o Trainee
2) What are the proactive measures and to what extent are they important in the success of B40 youth training?	o What are the challenges faced in B40 youth training and what are your action plans? o From a strategic perspectives, what is your strategic plan in order to maximise benefits and minimise challenges of training programme, upskilling, reskilling or employment, or entrepreneurship to the B40 youth especially? o What are the interests of the B40 youth group? o What kind of training do the B40 youth group want/need? o How do you identify and pool these B40 youth participants? o In your framework/governance, is there any particular emphasis on B40 youth?	o Trainer o Trainee o Policy makers

All interviews were transcribed word-for-word manually while maintaining the anonymity of interviewees. Pseudo names were assigned to each participant while categorising them in their respective stakeholder group. Accordingly, policymakers were referred to as PolicyMaker_1, PolicyMaker_2, PolicyMaker_3, and PolicyMaker_4; training providers’ were referred to as Trainer_1, and Trainer_2; and trainees were referred to as Trainee_1, Trainee_2, Trainee_3, Trainee_4 and Trainee_5. All transcriptions were coded using NVivo software with occasional word cloud queries based on the question type for a thematic analysis method. ^5^ Verbatim statements were also chosen for several questions through the software. The third researcher conducted the analysis using the software along with the first researcher’s contextual input. The third researcher proceeded to make inferences based on the analysed data. Member checking was conducted during and post interviews. ^7^ Answers to questions were repeated for confirmation from interviewees and findings of the report were shared with the interviewees. None of the interviewees suggested amendments to the data or findings reported. As detailed in Table 1, several validity and reliability measures for qualitative research studies were followed. ^7^

## Results

Each interview began with a brief demographic check. Details of the participants’ designations and roles are outlined in Table 3.

**Table 3.** Demographic details of interview participants.

Participant	Role description
PolicyMaker_1	o Strategic initiative department o Provides training opportunities for registered and non-registered employers o Have funding for SMEs, B40 groups, and entrepreneurs o Traces study/output assessments post-training o Funds for B40: entrepreneurship, employment, reskilling and upskilling
PolicyMaker_2	o Training placement centre department o In charge of employment opportunities o Implements various initiatives based on funding and various target groups o Works together with strategic initiative department
PolicyMaker_3	o Vice president for SME accounts o Customer outreach o Provides consultation and advisory towards SME employers in central region regarding training requirements
PolicyMaker_4	o Strategic division o Similar pillar of strategic initiative department
Trainer_1	o Works with women and youth in training provision o Provides training with certificates approved by ministry of human resources o Mostly entrepreneurship development programmes on marketing, digital marketing, financial literature, unique selling point, how to sell products, specific skills sharpening
Trainer_2	o Manager of training institute under human resources ministry o Provides diploma in computer networking and certificates for electrical level 1 to 5 (advanced diploma) o Collaborating with Malaysian Qualifications Agency to allow level 4 students to join university degree (technical and vocational education and training- TVET).
Trainee_1	o Completed diploma in training institute o Worked (practical) in training institute for 3 months post-training completion o Degree in IT network o Working in private company under IT networking site as project engineer o Youth
Trainee_2	o Studied wiring in training institute o Working in training college as a wiring teacher o Worked with father’s business post SPM (secondary school completion cert) o Youth
Trainee_3	o Studied level 1 to level 3 diploma in training institute o Working as technician leader o Attended air conditioner services on wiring course o Youth
Trainee_4	o Studied electric level 2 to level 4 in training institute o Worked as assistant teacher in training institute o Working as stock keeper currently o Youth
Trainee_5	o Studied level 2 and 3 wiring in training institute o Worked as technician o Studied further, waiting for cert o Started own business on house wiring, air conditioner services. o Youth

**Figure 1.**
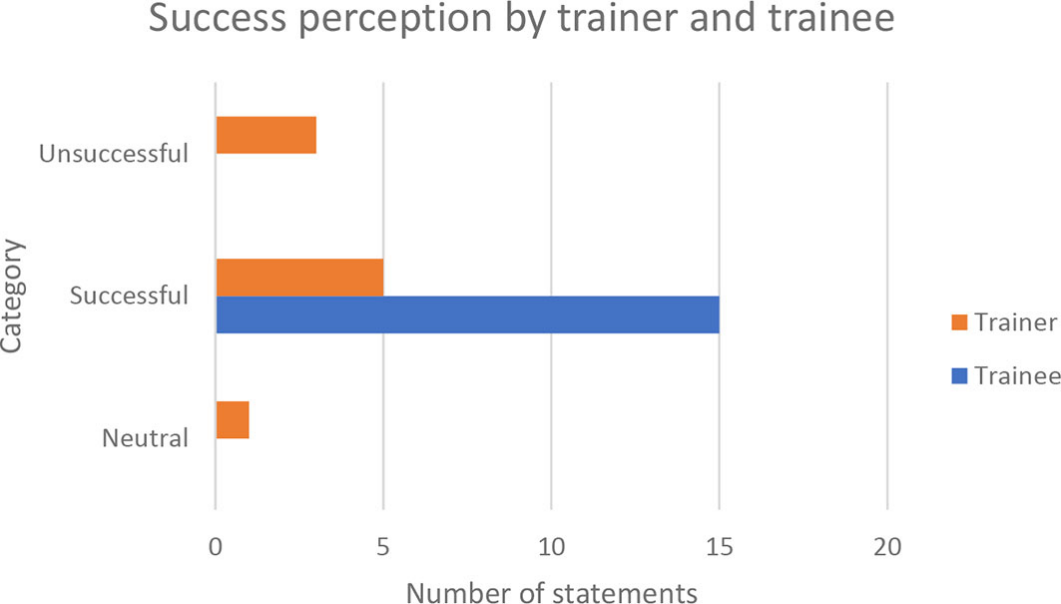
Training success perception by trainer and trainees.

Concerning research question 1 on the importance of training in the upliftment of B40 youth talents in line with poverty eradication, findings from the interviews are summarised in Figure 1. ^12^ Upon assigning sentiment codes to the transcriptions using NVivo, the majority of trainees and training providers were found to have attested to training being successful in uplifting B40 youth, thereby implying the importance of training in eradicating poverty through B40 youth upliftment.

**Figure 2.**
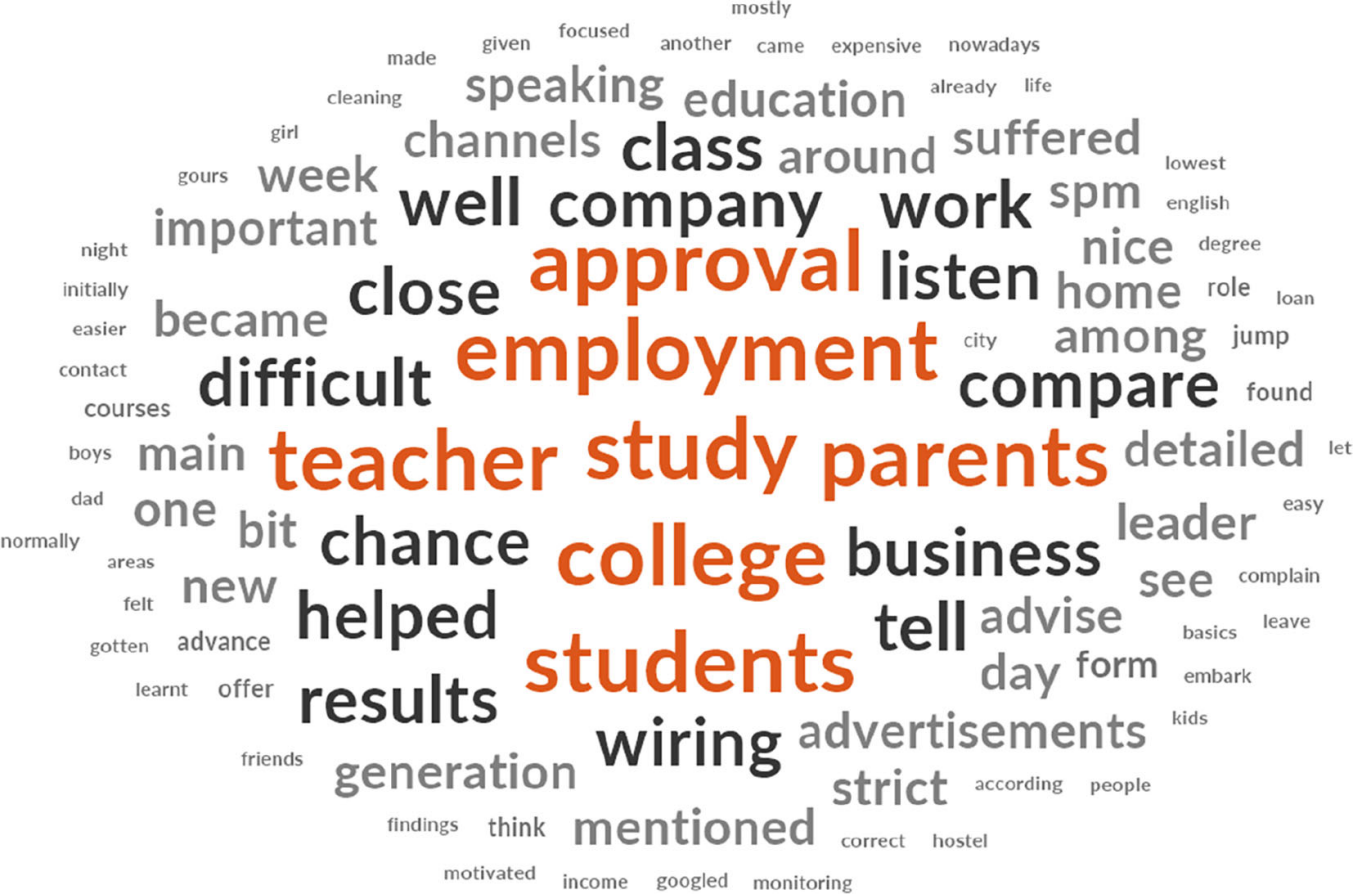
Word cloud diagram illustrating the key challenges faced by policymakers, training providers, and trainees.

**Figure 3.**
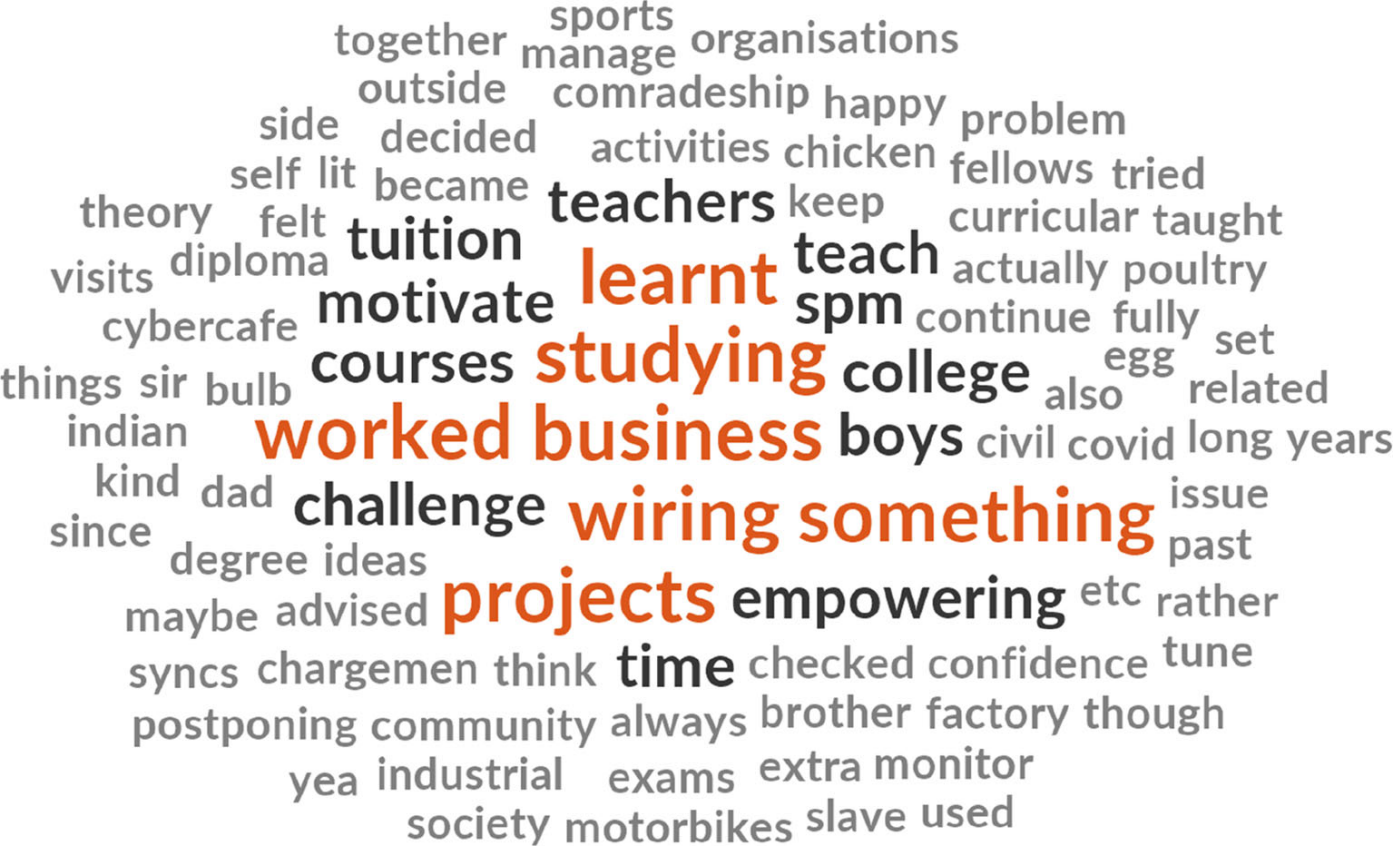
Word cloud diagram illustrating the key interests of the B40 youth.

As to research question 2 on the proactive measures and their crucial value in the upliftment of B40 youth to eradicate poverty, six themes were explored and summarised in their respective Tables and/or Figures. Firstly, on challenges faced in B40 youth training and action plans. Figure 2 shows that approval, employment, teacher, study, parents, college, and students are keywords used in explaining the challenges faced in B40 youth training. The initial 137 items with synonyms themes that emerged were reduced to 111 items after the removal of non-thematic words. Policymakers believed that tracking employment of the B40 youth after the approval of the training programmes for training providers would be particularly challenging given that the B40 youth tend to leave their jobs within one or two weeks and are very much into business opportunities. The training providers find it challenging to obtain the students’ focus for around eight hours a day while facing complaints from parents about their children being back home late at night. However, they believe that a close relationship with the students, close monitoring and constant updates to parents tend to keep these issues at bay. Meanwhile, trainees find it difficult to understand lessons taught by their teachers and would be required to google further explanations because their tutor pushes them to do so. They also face challenges from their parents not in favour of continuing studies or undergoing training because it would take up time that could be used to earn income at random jobs. Secondly, on interests of the B40 youth group. Figure 3 highlights certain themes that emerge including learning, business, wiring works, hands-on project, teaching, and studying. The initial 117 items with synonyms themes that emerged were reduced to 91 items post-removal non-thematic words. Third, regarding types of training wanted or needed by the B40 youth group (refer to Table 4). Fourth, measures to identify and pool B40 youth participants for training (refer to Table 5). Fifth, the emphasis in governance framework on B40 youth (refer to Table 6). Sixth, strategic perspectives on strategic plans to maximise benefit and minimise challenges of B40 youth training programmes for employment, entrepreneurship, reskilling and upskilling (refer to Table 7).

**Table 4.** Types of training wanted or needed by B40 youth.

Participant	Type(s) of training suggested
Trainer_1	o Professionalise ordinary jobs like tow truck o Soft skills
Trainee_1	o Positive thinking and attitude o Self-motivation o Self-improvement
Trainee_3	o Customer relationship
Trainee_5	o Detailed wiring courses

**Table 5.** Measures to identify and pool B40 youth for training.

Participant	Measures to pool B40 youth
PolicyMaker_3	o Set requirements o Household income group less than RM3,800 based on guideline set by nation o Training provider find participants o Policy makers request documents like ‘bantuan prihatin nasional’ (scheme for B40 household groups) before approval of training for those participants
PolicyMaker_2	o Collaborate with school counsellors o Promote in social media o Collaboration with technical colleges (TVET)
Trainer_1	o Identify location, community, select the participants o Do background check in their houses o Household income less than RM3,500
Trainer_2	o Collaborate with school counsellors o Promote in social media o Collaboration with technical colleges (TVET)

**Table 6.** Selected excerpts on governance framework targeted for B40 youth. COVID = coronavirus disease 2019.

Participant	Verbatim excerpts	Key Findings
PolicyMaker_1	“Our training programmes are not for the specific group … we receive some amount of funding to help the B40 group. So, we will look into the outcome first … they want to be employed in a company or... to be upskilled or reskilled … get business opportunities for them to generate own income.”	o There is no governance framework specifically targeted at the b40 group. o Training programmes are planned based on prospective outcome in line with government aspirations only when funding is received.
PolicyMaker_4	“..PENJANA. We received grant to cater to those affected during COVID... we don’t have specific scheme for them (B40 youth)... but we have B40 development to cater to B40... whenever training provider provide or propose their proposal, they need to propose the outcome as well... We are going to launch janapreneur-online business platform to participate as seller. Just focusing on B40 development”	o There is no governance framework specifically targeted at the B40 group. o B40 development funds can be utilised when proposed outcome from training provider is approved. o Future plans to be aimed at B40 development.

**Table 7.** Selected excerpts on strategic plans to maximise benefits of B40 youth trainings. COVID-19 = coronavirus disease 2019.

Participant	Verbatim excerpts	Key findings
PolicyMaker_1	“Our focus is very much driven by aspiration of government.. the aspiration … main goals for the funding and then we develop a plan afterwards … refers to the needs of the policy makers, government and then we also in future, we have plans to do certain target groups … B40 kind of trainings has been parked under other ministries.”	o Policies and strategic initiatives are driven by government aspirations. o Ongoing discussion on future policies to be targeted at certain groups like B40. o B40 trainings under other ministries.
PolicyMaker_2	“look into new employment. That is very crucial for now, they can be B40, they can be anyone... Many lost jobs during pandemic, but in future, there is demand for around 10K new jobs to be created … help the people to be future ready … B40, M40, or T20 …”	o Focus more on employment opportunities for anyone, not just B40 as many have lost jobs in times of COVID-19. o Future ready employment post COVID-19.

## Discussion and conclusion

Following the research questions and findings reported, this study makes the following propositions:

1) Training is the key to uplifting the B40 youth and eradicate poverty.
2) Proactive measures are imperative in the success of B40 youth training towards poverty eradication.

**Figure 4.**
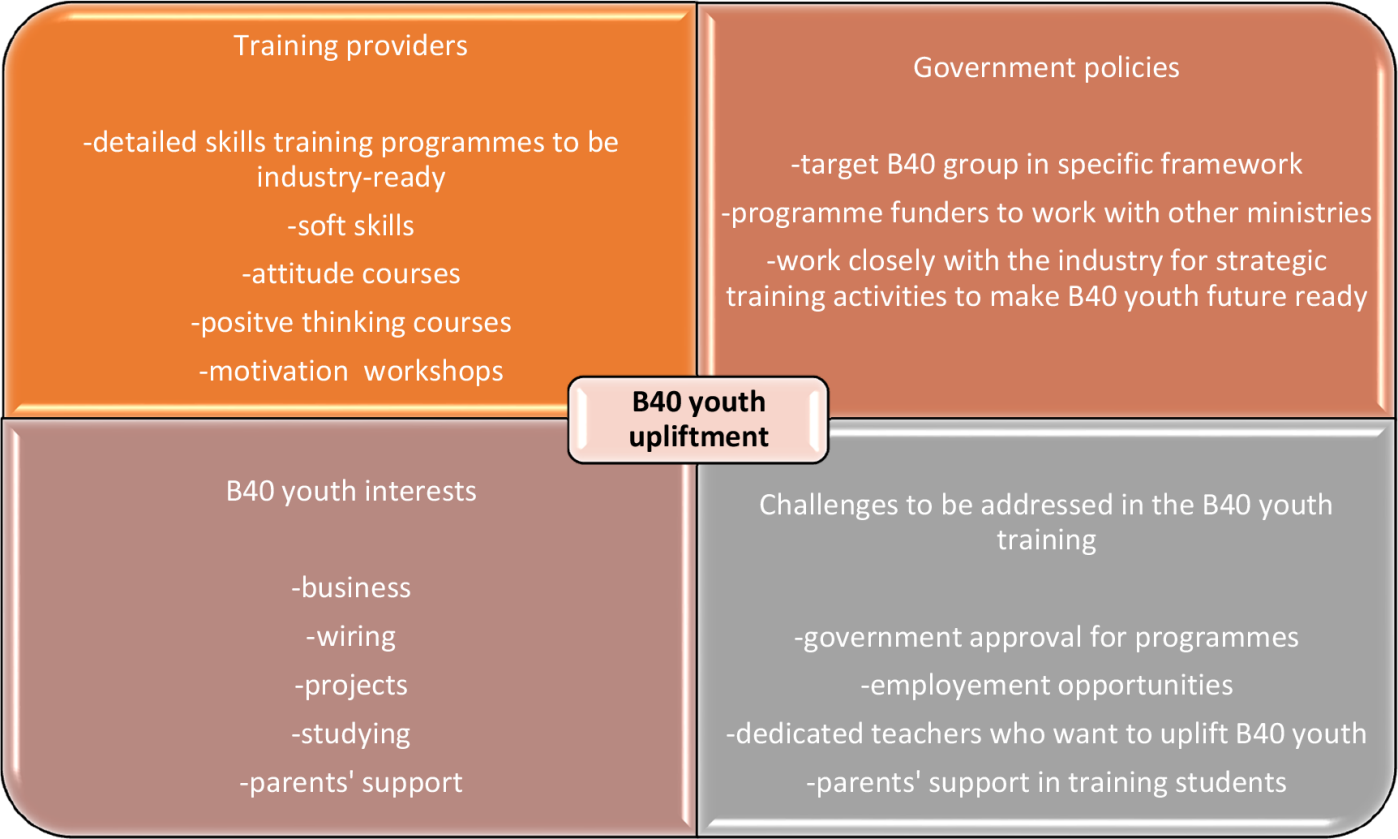
Framework for multi-stakeholder involvement towards the upliftment of B40 youth.

Although government aspirations under the shared prosperity vision 2030 have been set to uplift the B40 group, in practice, government training funders are not being guided by a specific framework for B40 youth development. Ministries in charge of B40 training need to work in collaboration with the funder, policymakers, and training providers to better attract the interest of the B40 youth and overcome challenges faced in their training. Figure 4 outlines a framework proposed by the researchers in which every stakeholder needs to come together to uplift B40 youth while considering their interests and the challenges that arise from the training journey. The findings of the study are congruent with the inclusive talent development practices that have been highlighted in previous studies. ^8^^,^^10^ The researchers conclude that through proper training and proactive measures via collaboration pathways of multi-stakeholder involvement, the gap between Malaysia’s aspirations and its actual practice on the ground can be closed. The training activities should also involve digital technology skills to keep abreast with the demands in this IR4.0 era.

The study contributes to the existing literature and practitioners by highlighting the current gap in Malaysia’s aspirations of eradicating poverty in their shared prosperity vision 2030 versus actual organisational practice. Thus, the study suggests that stakeholders come up with proactive strategies to ensure that the right trainees are matched with the right training providers and government policies. A linkage between government policies and industry requirements needs to be established as opposed to the present discontinuity. A structured training needs analysis plan should be put in place through collaborations between industries and governments. However, organisations should be empowered to make decisions instead of constant government reliance to ensure continuous professional development for all within the organisation is embraced. Moreover, B40 individuals commonly found in lower-level positions can be pooled into the career pathway towards a shift into M40. However, the unavailability of statistical representation disables the generalisability of this study’s findings. The qualitative nature of the study is subject to difficulty in causality investigation. Self-reported data are also subject to biases in subjective measures and social desirability.

## Data availability

### Underlying data

Figshare: Interview transcript with multi-stakeholder on training in Malaysia for poverty eradication. https://doi.org/10.6084/m9.figshare.16384770. ^12^

This project contains the underlying data:

• Compiled transcripts.pdf. (This file consists transcription of interview recordings conducted with Malaysia’s multi-stakeholder group including policy makers, trainers, and trainees to gauge proactive measures to eradicate poverty in Malaysia via training.)

### Extended data

Figshare: Interview guide for proactive measures to eradicate poverty in Malaysia in IR4.0 Era: A shared prosperity vision. https://doi.org/10.6084/m9.figshare.16735426. ^13^

This project contains the underlying data:

• Interview guide.pdf. (This file consists guide used during online semi-structured interviews.)

Data are available under the terms of the Creative Commons Attribution 4.0 International license (CC-BY 4.0).
