# Supplementary material for: Proactive measures to eradicate Malaysia’s poverty in IR4.0 era: a shared prosperity vision
Source: F1000Res. 2022 Jan 7;10:1094. Originally published 2021 Oct 28. [Version 2] doi: 10.12688/f1000research.73330.2 (PMC8790706; doi:10.12688/f1000research.73330.2)
Supplement: Peer review history

# Peer review: Reviewer response for version 2

**DOI:** 10.5256/f1000research.113688.r118905

**Published:** 2022-01-25

**Authors:** Lew Tek-Yew

**Affiliations:** 1 Faculty of Business, Curtin University Malaysia, Miri, Malaysia

This paper can now be accepted for indexing.

Is the work clearly and accurately presented and does it cite the current literature?

Partly

If applicable, is the statistical analysis and its interpretation appropriate?

Yes

Are all the source data underlying the results available to ensure full reproducibility?

Yes

Is the study design appropriate and is the work technically sound?

Yes

Are the conclusions drawn adequately supported by the results?

Partly

Are sufficient details of methods and analysis provided to allow replication by others?

Yes

Reviewer Expertise:

NA

I confirm that I have read this submission and believe that I have an appropriate level of expertise to confirm that it is of an acceptable scientific standard.

# Peer review: Reviewer response for version 1

**DOI:** 10.5256/f1000research.76974.r98149

**Published:** 2021-12-13

**Authors:** Magda L. Hewitt

**Affiliations:** 1 University of Johannesburg, Johannesburg, South Africa

The purpose of the study is clearly stated. It is relevant by exploring the mismatch (assumption) between strategic government intervention in managing B40 talent in the IR4.0 era. The chosen methodology is well described and thoroughly executed and reported on. Not clear what is meant by ''very much into business opportunities". The sample of trainees might be too small to draw any inferences and the needs of the type of training required by them, reflect personal needs. It would be interesting to know how this relates to national training needs. Interesting findings are the training hours and learning material content. This needs more interrogation. The challenges faced by the B40 group are insightful and their reality should be considered when thinking about the study suggestions. 

 I missed a brief overview of 4IR.0 context within this study.

Is the work clearly and accurately presented and does it cite the current literature?

Yes

If applicable, is the statistical analysis and its interpretation appropriate?

Not applicable

Are all the source data underlying the results available to ensure full reproducibility?

Yes

Is the study design appropriate and is the work technically sound?

Yes

Are the conclusions drawn adequately supported by the results?

Yes

Are sufficient details of methods and analysis provided to allow replication by others?

Yes

Reviewer Expertise:

Entrepreneurship, emerging economies, leadership; entrepreneurial leaderhsip

I confirm that I have read this submission and believe that I have an appropriate level of expertise to confirm that it is of an acceptable scientific standard.

# Peer review: Reviewer response for version 1

**DOI:** 10.5256/f1000research.76974.r98146

**Published:** 2021-11-30

**Authors:** Lew Tek-Yew

**Affiliations:** 1 Faculty of Business, Curtin University Malaysia, Miri, Malaysia

The article addresses a very important topic of ensuring that training provided by the trainers based on funding from the government has met the objectives of alleviating the poverty level of the B40 youth in Malaysia. The qualitative method used is appropriate for exploratory study.  Conclusions are based on the results of the study.  

 The authors need to cite the latest literature published in 2020 and 2021 to justify the relationship between training and poverty eradication.

 The discussion and conclusion section needs to be elaborated to provide the answers to each of the research questions especially on the issue of ‘To what extent: -

*This study aims to explore the rationale of government intervention in managing B40 youth talent via training in this IR4.0 era. The following research questions guide the study. Research question 1: To what extent is training important in uplifting the B40 youth in line with poverty eradication? Research question 2: What are the proactive measures and to what extent are they crucial to the success of B40 youth training towards poverty eradication?*

Is the work clearly and accurately presented and does it cite the current literature?

Partly

If applicable, is the statistical analysis and its interpretation appropriate?

Yes

Are all the source data underlying the results available to ensure full reproducibility?

Yes

Is the study design appropriate and is the work technically sound?

Yes

Are the conclusions drawn adequately supported by the results?

Partly

Are sufficient details of methods and analysis provided to allow replication by others?

Yes

Reviewer Expertise:

NA

I confirm that I have read this submission and believe that I have an appropriate level of expertise to confirm that it is of an acceptable scientific standard, however I have significant reservations, as outlined above.

# Response

**Published:** 2021-12-16

**Authors:** Darshana Darmalinggam

**Affiliations:** University of Nottingham Malaysia, Malaysia

Dear respected reviewer, 

 We thank you for a chance to improve our paper. We have compiled the suggestions and comments as per your call, and have addressed the concerns based on your excellent suggestion as follows:-

1. It is a new area of research. As such, no direct citations can be found in 2020-2021.
2. The discussion and conclusion section has now been elaborated to provide answers to each of the research questions especially on the issue of 'To what extent training is important in uplifting B40 youth in line with poverty eradication?' and 'What are the proactive measures and to what extent are they crucial to the success of B40 youth training towards poverty eradication?'

Our deepest gratitude to all the Respected Editors and Reviewers for your valuable time taken to scrutinise the content of this paper. We have attempted our very best to improve the quality of this paper.  

 Thank you so much.
